# Relationship Between Nursing Diagnoses, Mortality and Length of Stay in the ICU: A Retrospective Cohort Study

**DOI:** 10.1111/nicc.70600

**Published:** 2026-08-05

**Authors:** José Carlos de Santana Neto, Paulo Carlos García, Tatiane Martins de Matos, Karina Sichieri, Diná de Almeida Lopes Monteiro da Cruz

**Affiliations:** ^1^ Postgraduate Program in Nursing in Adult Health School of Nursing of the University of São Paulo São Paulo Brazil; ^2^ Medical‐Surgical Nursing Department School of Nursing of the University of São Paulo São Paulo Brazil; ^3^ Clinical Nursing Division, Nursing Department University Hospital of the University of São Paulo São Paulo Brazil

**Keywords:** critical care nursing, critical care outcomes, standardised nursing terminology

## Abstract

**Background:**

Nursing process is a systematic, problem‐solving framework structuring clinical reasoning and patient‐centred care; within it, Nursing Diagnoses (ND) link clinical assessment to planning and implementing individualised interventions. However, evidence demonstrating ND ability to predict clinical outcomes using severity indices remains lacking.

**Aims:**

The primary aim of this study was to describe the relationship between the number of ND on admission to an Intensive Care Unit (ICU) with mortality and ICU length of stay (ICU‐LOS). Secondarily, we aimed to assess their association with Charlson comorbidity index (CCI), Simplified Acute Physiology Score III (SAPS‐3) score and Nursing activities score (NAS).

**Study Design:**

Retrospective cohort (22 April 2021–22 April 2022) study using data from medical records of patients aged ≥ 15 years admitted to an adult ICU of a Brazilian University Hospital. ND were based on the NANDA‐International taxonomy. According to the distributions of the variables, statistical tests were used to verify the relationship between the frequency of ND and the variables of interest.

**Results:**

The sample (*n* = 904; 54.4% male) had a mean age of 62.3 years (range: 15–101) and an average ICU‐LOS of 6.2 days (range: 1–79). There was an average of 5.2 ND per admission. No statistically significant relationship was observed between the number of ND and ICU‐LOS, CCI or SAPS‐3. However, logistic regression showed that each additional ND increased the odds of death by 13.6% (OR = 1.136; 95% CI: 1.035–1.248). Additionally, linear regression indicated a 0.65 percentage points NAS increase per additional ND (approximately 9 additional minutes of nursing care per day).

**Conclusion:**

The number of ND at ICU admission is an independent predictor of mortality and nursing workload.

**Relevance to Clinical Practice:**

These results support the clinical utility of ND, reinforcing their importance for standardising nursing data in practice and research.

## Impact Statements

- What is known about the topic
  ○ Standardised nursing diagnoses (ND) are essential components of the nursing process, utilised to plan and document patient‐centred care and improve communication within healthcare teams.
  ○ Previous evidence in general hospital settings indicates that the number and type of ND have a predictive capacity for clinical outcomes, including mortality, length of stay and nursing workload.
  ○ Despite their established use, high‐quality studies exploring the association between ND and specific Intensive Care Unit (ICU) severity indices, such as SAPS‐3, remain scarce.
- What this paper adds
  ○ This study establishes the number of nursing diagnoses at ICU admission as an independent predictor of mortality, showing that each additional diagnosis increases the odds of death by 13.6%.
  ○ It demonstrates that nursing workload is directly influenced by the quantity of nursing diagnoses, regardless of the patient's clinical severity or age.
  ○ The results reinforce the importance of standardised nursing language for risk identification and human resource management in high‐intensity care environments.

## 1 Introduction/Background

Nursing Process (NP) is established as a guiding model for nursing care practice, comprising five sequential and cyclical stages: assessment, diagnosis, planning, implementation and evaluation [1, 2, 3]. From the assessment stage, nurses interpret clinical data as nursing diagnoses (ND) related to patient problems, risks and syndromes.

Although ND are believed to be related to health outcomes, evidence supporting this association remains limited. Demonstrating that ND can be linked to hospital outcomes can highlight their importance not only clinically but also for resource allocation, treatment planning and health management [4, 5, 6, 7].

Taxonomies such as NANDA‐International (NANDA‐I), Omaha System and International Classification for Nursing Practice standardise ND language, improving nursing team communication, care quality, nursing process evaluation, nursing visibility and adherence to care standards [8, 9, 10].

D'Agostino et al. [6] and Sanson et al. [7] demonstrated predictive capacity of ND for outcomes such as length of stay (LOS), mortality, quality of life and nursing workload. However, these studies were not conducted in ICU settings and did not use ICU‐specific clinical severity indices such as Simplified Acute Physiology Score III (SAPS‐3). Internationally, the use of standardised ND in ICU remains heterogeneous, varying across countries, institutions and nursing education systems, with adoption more consolidated where electronic nursing documentation systems are established [10, 11].

To the best of our knowledge, no study has examined the relationship between the number of ND at ICU admission and mortality, LOS, clinical severity, comorbidity burden and nursing workload, using ICU‐specific indices. This gap limits understanding of the clinical and managerial utility of ND in high‐complexity settings.

### 1.1 Aims

This study aimed to analyse the relationship between the number of ND at admission to an Adult ICU (A‐ICU) with mortality and ICU length of stay (ICU‐LOS). As secondary objectives, we aimed to evaluate its association with Charlson comorbidity index (CCI), clinical severity according to the SAPS‐3 and nursing workload measured by Nursing activities score (NAS).

## 2 Methods

### 2.1 Design

This was a secondary analysis of a cohort dataset [12] comprising routine clinical data extracted from medical records of patients admitted to an A‐ICU of a secondary‐level university hospital, reported following Strengthening the Reporting of Observational Studies in Epidemiology guidelines [13] (Data S1).

### 2.2 Setting

The primary study was conducted in a mixed A‐ICU of a public secondary‐level university hospital in Brazil, with 12 active beds providing intensive care for clinical and surgical patients aged ≥ 15 years, operating on an approximately 1:1.5 nurse‐to‐patient ratio. The unit follows a primary nursing organisational model, in which each nurse (all holding a bachelor's degree) is assigned to fixed beds on a fortnightly rotation across shifts, being responsible for conducting the nursing assessment, establishing the nursing care plan and formulating ND for their patients.

### 2.3 Participants

The study population comprised patients from a database developed in a previous primary study [12], which initially included 940 admissions over an 18‐month period.

Patients aged ≥ 15 years of both sexes were eligible. Exclusions comprised patients admitted for specific procedures, those with LOS < 24 h, those aged < 15 years and those without ND properly recorded at admission.

### 2.4 Outcomes and Measures

The primary predictor variable was the number of ND at A‐ICU admission, obtained from the PROCEnf‐USP electronic system [14], which supports decision‐making and documentation of nursing process using standardised NANDA‐I diagnoses [15]. In daily clinical routine, ND are established by the responsible nurse upon admission following systematic assessment (anamnesis, physical examination and medical record review), typically documented within 2 h of admission, once the patient has been stabilised and settled, and re‐evaluated daily or upon clinical changes. However, the present study exclusively analysed the ND recorded at the time of A‐ICU admission.

Primary outcomes were ICU mortality (death during the ICU stay) and ICU‐LOS (days from admission to discharge), both extracted from the PROCEnf‐USP system. Secondary measures were:

- Clinical severity: Assessed upon the A‐ICU admission using SAPS‐3, a prognostic score calculated from 20 variables (including patient characteristics, admission context and acute physiological derangements), estimating in‐hospital mortality risk [16], completed by the admitting physician;
- Comorbidity burden: Evaluated using CCI, which assigns weights to 19 comorbid conditions, such as diabetes, malignancies and organ failures, combined with an age‐based increment, to estimate long‐term prognostic risk [17], completed by the responsible nurse;
- Nursing workload: Measured by NAS [18], a validated 23‐item instrument with seven categories (Basic activities, Ventilatory support, Cardiovascular support, Renal support, Neurological support, Metabolic/endocrine support and Specific interventions), in which scores express the percentage of a nurse's time dedicated to a patient over a 24 h (range: 0%–176.8%), widely used in ICU worldwide [19, 20, 21]. NAS was completed by the responsible nurse based on all nursing activities required by the patient, regardless of who performed them. Only the first 24‐h score was used.

### 2.5 Data Collection

The dataset was constructed by linking two sources via patient's hospital registration number: (1) the Garcia et al. [12] database, providing ND, sociodemographic data, admission and discharge dates, and discharge modality from PROCEnf‐USP; and (2) routine clinical monitoring spreadsheets, from which SAPS‐3, CCI and NAS were retrieved. When scores were absent, they were retrospectively calculated from medical records by the research team, following original validated algorithms, with no statistical imputation. The final dataset contained no missing data.

### 2.6 Statistical Analysis

Data were analysed using R software, version 4.3.2 [22]. Categorical variables were described through absolute (*n*) and relative (%) frequencies; continuous variables as mean, standard deviation (SD) and range. Normality of continuous variables was assessed through visual inspection of Q‐Q plots.

Pearson's correlation (r) was calculated for the number of ND versus ICU‐LOS, NAS, SAPS‐3 and CCI. For mortality, the Brunner‐Munzel test compared the number of ND between survivors and non‐survivors (transferred to any ward, transferred to another healthcare service or discharged home).

Regression models included binary logistic regression (mortality), negative binomial generalised linear model (ICU‐LOS) and linear regression (NAS), all adjusted for number of ND, age, sex and SAPS‐3.

Significance was set at 5%; results were presented with a 95% confidence interval (CI). An independent institutional statistician performed all analyses.

### 2.7 Ethical and Institutional Approvals

The original dataset was derived from the primary study by Garcia et al. [12], conducted under its own ethical approval. As this study required the collection of additional variables not available in the original database (NAS, SAPS‐3 and CCI), a new ethical approval was obtained from the Research Ethics Committee of the Hospital Universitário da USP (Approval Number: 6.760.637, CAAE: 78295224.9.0000.0076) on 12 April 2024, with data collection carried out immediately thereafter. A waiver of informed consent was granted by the ethics committee given the secondary analysis design. Data were de‐identified in compliance with institutional guidelines.

## 3 Results

Of 940 admissions occurred during the 18‐month study period (22 April 2021 to 22 October 2022), 36 were excluded: one aged < 15 years old, two admitted for specific procedures (bedside bronchoscopy and electric cardioversion), one with incomplete ND records and 32 with ICU‐LOS < 24 h, yielding a final sample of 904 admissions.

Patient characteristics and the ND frequencies are described in Table 1. Most admissions involved patients aged ≥ 60 years (61.84%; *n* = 559), with predominance of male patients (54.42%; *n* = 492). Mean ICU‐LOS was 6.19 days (SD 7.41; maximum 79 days). The main admission source was the operating theatre (46.9%; *n* = 424), followed by the accident and emergency department (32.63%; *n* = 295). Most patients were discharged to the ward (surgical unit, medical unit or rooming‐in; 77.32%; *n* = 699), while 18.03% (*n* = 163) died during the A‐ICU stay. Mean SAPS‐3 was 50.44 (SD 16.82), mean NAS was 65.97% (SD 17.02%; equivalent to 15.83 daily nursing hours) and mean CCI was 2.94 (SD 2.36).

**TABLE 1:** Main characteristics of the studied population (*n* = 904).

Data	Mean (SD)	Range	Frequency (%)	95% CI
Age	62.34 (18.71)	15–101	—	(61.10–63.54)^a^
Sex				
Male	—	—	492 (54.42)	(51.17–57.65)^b^
Female	—	—	412 (45.58)	(42.35–48.83)^b^
Length of stay	6.19 (7.41)	1–79	—	(5.75–6.72)^a^
Discharge mode				
Discharge to surgical unit	—	—	454 (50.22)	(46.97–53.47)^b^
Discharge to medical unit	—	—	232 (25.66)	(22.92–28.61)^b^
Death	—	—	163 (18.03)	(15.66–20.67)^b^
Transfer to another healthcare service	—	—	29 (3.21)	(2.23–4.59)^b^
Discharge to rooming‐in	—	—	13 (1.44)	(0.82–2.47)^b^
Discharge to home	—	—	13 (1.44)	(0.82–2.47)^b^
Number of nursing diagnoses	5.19 (2.06)	1–14	—	(5.06–5.33)^a^
Survivors	5.02 (1.92)	1–12	—	(4.88–5.16)^a^
Deaths	5.99 (2.49)	1–14	—	(5.63–6.39)^a^
SAPS‐3	50.44 (16.82)	14–118	—	(49.36–51.55)^a^
NAS	65.97 (17.02)	28.1–135.8	—	(64.88–67.10)^a^
CCI	2.94 (2.36)	0–14	—	(2.79–3.10)^a^

Abbreviations: CCI, Charlson comorbidity index; CI, confidence interval; NAS, nursing activities score; SAPS‐3, simplified acute physiology score III; SD, standard deviation.

^a^ ABC method.

^b^ Agresti‐Coull method.

Nurses identified a mean of 5.19 ND per admission (SD 2.06; range: 1–14), with 81 distinct ND recorded. The most prevalent was Risk for infection (00004), present in 80.64% (*n* = 729) of admissions. Table 2 shows the frequency of the ND with a frequency greater than 5%; complete data regarding frequency of ND is available on Table S1.

**TABLE 2:** Frequency of the nursing diagnoses identified with a frequency greater than 5% (*n* = 904).

Nursing Diagnosis	Frequency (%)	95% CI
Risk for infection (00004)	729 (80.64)	(77.94–83.09)^a^
Risk for adult pressure injury (00304)^b^	433 (47.89)	(44.66–51.16)^a^
Acute pain (00132)	344 (38.05)	(34.94–41.26)^a^
Impaired tissue integrity (00044)	322 (35.62)	(32.56–38.80)^a^
Risk for falls (00155)	321 (35.51)	(32.46–38.68)^a^
Risk for aspiration (00039)	301 (33.29)	(30.30–36.43)^a^
Risk for unstable blood glucose (00179)	247 (27.32)	(24.52–30.32)^a^
Decreased cardiac output (00029)	231 (25.55)	(22.82–28.5)^a^
Bathing self‐care deficit (00108)	187 (20.69)	(18.17–23.45)^a^
Risk for venous thromboembolism (00268)	133 (14.71)	(12.55–17.18)^a^
Ineffective breathing pattern (00032)	123 (13.61)	(11.52–16.00)^a^
Toileting self‐care deficit (00110)	113 (12.50)	(10.50–14.82)^a^
Ineffective peripheral tissue perfusion (00204)	109 (12.06)	(10.09–14.35)^a^
Risk for bleeding (00206)	95 (10.51)	(8.67–12.68)^a^
Ineffective tissue perfusion (specify type: renal, cerebral, cardiopulmonary, gastrointestinal, peripheral) (00024)	87 (9.62)	(7.86–11.73)^a^
Impaired spontaneous ventilation (00033)	87 (9.62)	(7.86–11.73)^a^
Acute confusion (000128)	86 (9.51)	(7.76–11.61)^a^
Impaired gas exchange (00030)	65 (7.19)	(5.67–9.07)^a^
Ineffective protection (00043)	62 (6.86)	(5.38–8.71)^a^
Risk for imbalanced fluid volume (00025)	61 (6.75)	(5.28–8.58)^a^
Impaired skin integrity (00046)	49 (5.42)	(4.11–7.11)^a^
Risk for electrolyte imbalance (00195)	48 (5.31)	(4.02–6.98)^a^

Abbreviation: CI, confidence interval.

^a^ Agresti‐Coull method.

^b^ Some patients were diagnosed with ‘Risk for pressure ulcer (00249)’ and others with ‘Risk for adult pressure injury (00304)’, the latter being the updated term in the 12th edition of the NANDA‐I classification (2021–2023). For this study, both diagnoses were considered equivalent and analysed as a single diagnosis.

Patients who died had a higher mean number of ND per admission (5.99, SD 2.49) compared to survivors (5.02, SD 1.92). Brunner‐Munzel test confirmed a statistically significant difference between groups (B = −4.534; *p* < 0.001). Logistic regression showed that each additional ND increased the odds of death by 13.6% (OR: 1.136; 95% CI: 1.035–1.248; *p* = 0.007); each additional year of age or SAPS‐3 point increased odds by 1.7% and 7.8%, respectively. Sex was not statistically associated with mortality (Table 3).

**TABLE 3:** Logistic regression of mortality as a function of the number of nursing diagnoses and other variables.

	OR	Standard Error	95% CI	VIF	*p*
Data					
Intercept	0.001	0.000	(0.000–0.002)	—	**< 0.001**
Number of ND	1.136	0.054	(1.035–1.248)	1013	**0.007**
Age	1.017	0.007	(1.004–1.030)	1043	**0.010**
Sex (Ref: Female)	1.000				
Sex (Male)	0.950	0.194	(0.637–1.419)	1036	0.802
SAPS‐3	1.078	0.007	(1.064–1.094)	1009	**< 0.001**
Model metrics					
AUC	0.834		(0.798–0.870)		
Pseudo‐*R* ^2^ (McFadden)	0.262				
Omnibus test	204.97				**< 0.001**
Hosmer–Lemeshow goodness‐of‐fit test	11.975				0.152

*Note:* Bold values indicate statistical significance (*p* < 0.05).

Abbreviations: CI, confidence interval; ND, nursing diagnoses; OR, odds ratio; SAPS‐3, simplified acute physiology score III; VIF, variance inflation factors.

**FIGURE 1:**
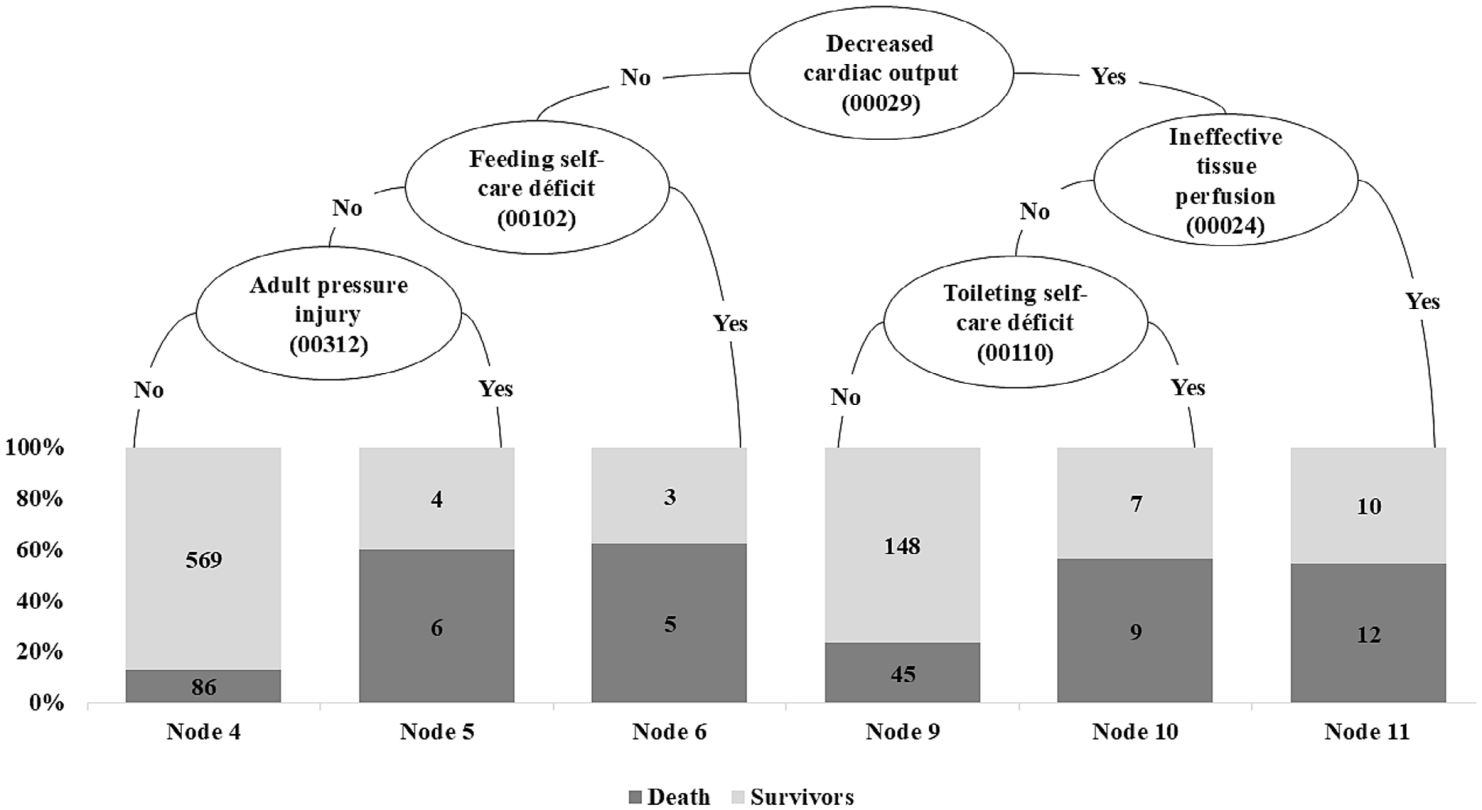
Classification and Regression Tree analysis with nursing diagnoses and mortality rates. Yes: They had the respective NANDA‐I diagnosis identified and recorded at the time of admission. No: They did not have the respective NANDA‐I diagnosis identified and recorded at admission.

A Classification and Regression Tree (CART) identified six groups of patients (Figure 1): two with lower mortality rates (13.13% and 23.32%) and four with high mortality, ranging from 54.55% to 62.5%. Model accuracy was 82.9% (specificity 96.08%; sensitivity 19.6%; cross‐validation 19.03%).

The number of ND showed low correlation with ICU‐LOS (*r* = 0.068; *p* = 0.040). The negative binomial generalised linear model found no statistically significant association between ND and ICU‐LOS (*p* = 0.072) or sex (*p* = 0.074). However, each additional year of age reduced ICU‐LOS by 0.4% (95% CI: 0.992–0.999; *p* = 0.009), and each SAPS‐3 point increased it by 1.7% (95% CI: 1.013–1.021; *p* < 0.001) (Table 4).

**TABLE 4:** Generalised linear model for negative binomial distribution of length of stay as a function of the number of nursing diagnoses and other variables.

	RR	SE	95% CI	VIF	*p*
Data					
Intercept	2.772	0.383	(2.076–3.708)	—	**< 0.001**
Number of ND	1.027	0.015	(0.998–1.058)	1.076	0.072
Age	0.996	0.002	(0.992–0.999)	1.100	**0.009**
Sex (Ref: Female)	1.000				
Sex (Male)	1.113	0.067	(0.990–1.252)	1.025	0.074
SAPS‐3	1.017	0.002	(1.013–1.021)	1.092	**< 0.001**
Model metrics					
Pseudo‐*R* ^2^ (Nagelkerke)	0.0875				
Omnibus test	82.535				**< 0.001**

*Note:* Bold values indicate statistical significance (*p* < 0.05).

Abbreviations: CI, confidence interval; ND, nursing diagnoses; RR, rate ratio; SAPS‐3, simplified acute physiology score III; VIF, variance inflation factors.

Low correlations were observed between number of ND and NAS (*r* = 0.113; *p* < 0.001), SAPS‐3 (*r* = 0.220; *p* < 0.001) and CCI (*r* = 0.149; *p* < 0.001). Linear regression showed that each additional ND independently increased NAS by 0.624 percentage points (95% CI: 0.102–1.147; *p* = 0.019); each additional year of age decreased NAS by 0.171 percentage points (95% CI: −0.229 to −0.113; *p* < 0.001); and each SAPS‐3 point increased NAS by 0.341 percentage points (95% CI: 0.277–0.406; *p* < 0.001). Sex was not statistically significant (Table 5).

**TABLE 5:** Linear regression of NAS as a function of the number of nursing diagnoses and other variables (*n* = 904).

	Coefficient (b)	SE	95% CI	VIF	*p*
Data					
Intercept	56.341	2.453	(51.526–61.156)	—	**< 0.001**
Number of ND	0.624	0.266	(0.102–1.147)	1078	**0.019**
Age	−0.171	0.030	(−0.229 to −0.113)	1100	**< 0.001**
Sex (Ref: Female)	0				
Sex (Male)	−0.309	1.075	(−2.418–1.800)	1026	0.774
SAPS‐3	0.341	0.033	(0.277–0.406)	1094	**< 0.001**
Model metrics					
*R* ^2^	0.132				
Adjusted *R* ^2^	0.129				
*F*‐statistics	34.28				**< 0.001**

*Note:* Bold values indicate statistical significance (*p* < 0.05).

Abbreviations: CI, confidence interval; NAS, nursing activities score; ND, nursing diagnoses; SAPS‐3, simplified acute physiology score III; VIF, variance inflation factors.

## 4 Discussion

This study examined the relationship between the number of ND identified at ICU admission and clinical and workload outcomes in 904 admissions to an adult ICU. The number of ND at admission was an independent predictor of ICU mortality and nursing workload, but not of ICU‐LOS, clinical severity or comorbidity burden.

The predominance of male and elderly patients in this A‐ICU is consistent with previous literature from Brazil [23], and may reflect sociocultural factors, lower adherence to health services and greater exposure to accidents and violence.

Nurses identified an average of 5.19 ND per patient, slightly higher than the 4.5 reported by D'Agostino et al. [24] in Italian medical and surgical inpatient units, reflecting the greater physiological instability and care demands of ICU patients. The most frequent ND was Risk for infection (00004), followed by Risk for adult pressure injury (00249/00304) and Acute pain (00132), consistent with D'Agostino et al. [11], an international review which identified Risk for infection as the most prevalent ND in nine of 10 ICU studies. The predominance of diagnoses from Domain 11—Safety/Protection and Domain 4—Activity/Rest reflects the acute care profile of ICU patients, as also observed by Sanson et al. [7] in an Italian ICU.

Each additional ND at admission increased the odds of ICU death by approximately 14%, after adjusting for age, sex and SAPS‐3. These results align with Bertocchi et al. [4], Castellan et al. [5] and Sanson et al. [7, 10], all conducted in Italy, reinforcing that a broader range of human responses captured by ND is associated with greater mortality risk. This suggests that ND reflect not only current care needs but also cumulative patient vulnerability, dimensions that conventional severity scores are not designed to capture, positioning ND as a complementary tool for early risk stratification in critical care.

CART analysis identified subgroups with notably high death rates, though with low sensitivity (19.6%) and a cross‐validation error of 19.03%, indicating limited generalisability. Despite these constraints, CART remains useful for identifying high‐risk subgroups requiring greater clinical attention [25].

No independent association was found between ND and ICU‐LOS, with ICU‐LOS primarily explained by age and SAPS‐3. This contradicts D'Agostino et al. [6, 24] and Sanson et al. [10], conducted in Italy, and may reflect clinical and organisational factors, such as the mixed clinical‐surgical case mix, the high proportion of post‐operative admissions and ward bed availability.

The mean SAPS‐3 of 50.44 confirmed good calibration of this score in this setting [26], with predicted mortality closely matching the observed rate of 18.03%. The absence of correlation between SAPS‐3 and the number of ND suggests these tools capture distinct dimensions of patient complexity: while SAPS‐3 reflects physiological derangement, ND represents the patient's immediate human responses. Similarly, the absence of correlation with CCI suggests that the number of ND in ICU primarily reflects acute rather than chronic care needs.

Finally, each additional ND independently increased NAS by 0.624 percentage points (approximately 9 min of nursing care per day), independent of SAPS‐3, age and sex, indicating that nursing workload is shaped not only by clinical severity but also by the care complexity captured by ND. This result should be interpreted cautiously, as it reflects a single admission assessment and NAS tends to decline over hospitalisation [27]. Beyond workload quantification, the systematic use of standardised nursing language, such as NANDA‐I, in electronic health records supports the generation of nursing‐sensitive outcome data, contributing to an international evidence base for nursing practice in critical care.

### 4.1 Strengths and Limitations

The large sample of 904 consecutive admissions with no missing data strengthens internal validity. The 18‐month data collection period mitigates seasonal variation effects, and the use of routinely recorded real‐world data enhances ecological validity. The PROCEnf‐USP system with standardised NANDA‐I taxonomy promoted consistency in ND recording, and multivariate analyses were performed by an independent statistician, reducing the risk of analytical bias.

Limitations included the possibility that PROCEnf‐USP may not reflect current NANDA‐I taxonomy updates, potentially leading to the recording of outdated diagnoses or overlooked newly added ones. Since completion of the system's diagnostic questionnaire is optional, ND identification may have varied across nurses, and diagnostic accuracy was not formally assessed. The primary nursing model adopted in the A‐ICU, with fortnightly bed rotation, may have introduced variability in documentation practices, and nurse‐level variables were not assessed in the present study. The retrospective single‐centre design precludes causal inference and limits generalisability.

### 4.2 Recommendations or Implications for Practice and/or Further Research

This study provides evidence for the use of ND as early prognostic indicators for mortality and as independent reflectors of nursing workload in the ICU, reinforcing their role in resource allocation and clinical decision‐making. Future multicentre, prospective and longitudinal studies are recommended to confirm and extend these results, explore causal relationships and assess the impact of ND on nursing workload throughout the ICU stay, as well as to refine predictive models and evaluate nurses' diagnostic accuracy with electronic decision‐support tools.

## 5 Conclusion

This study reinforces the importance of ND in critical care, demonstrating independent associations with mortality and nursing workload. These results highlight the role of ND in risk identification and clinical decision‐making, contributing to patient safety and care quality in high‐intensity environments. Further research in diverse clinical contexts is needed to broaden the understanding of these relationships.

## Author Contributions

**José Carlos de Santana Neto:** conceptualization, data collection, data analysis, methodology, project administration, writing original draft, writing review and editing. **Paulo Carlos García:** conceptualization, data collection, data analysis, study supervision, methodology, project administration, writing original draft, writing review and editing. **Tatiane Martins de Matos:** conceptualization and data curation. **Karina Sichieri:** conceptualization and data curation. **Diná de Almeida Lopes Monteiro da Cruz:** conceptualization, data analysis, study supervision, methodology, project administration, writing original draft, writing review and editing.

## Funding

This research was supported by the residency program of nursing in adult and elderly health, which is funded by the Ministry of Health. This study was financed in part by the *Coordenação de Aperfeiçoamento de Pessoal de Nível Superior—Brasil* (CAPES)—Finance Code 001. Cruz DALM holds a CNPq Productivity in Researcher scholarship (#316768/2021–9).

## Ethics Statement

Before data collection, the HU‐USP Research Ethics Committee granted ethical approval (Approval Number: 6.760.637, CAAE: 78295224.9.0000.0076) on 12 April 2024.

## Consent

The requirement for informed consent was waived by the ethics committee.

## Conflicts of Interest

The authors declare no conflicts of interest.

## Supporting information


**Data S1:** STROBE Checklist of items that should be included in reports of cohort studies.

**Table S1:** Frequency of the nursing diagnoses identified (*n* = 904).

## Data Availability

The data that support the findings of this study are available on request from the corresponding author. The data are not publicly available due to privacy or ethical restrictions.
