# Supplementary material for: Relationship Between Nursing Diagnoses, Mortality and Length of Stay in the ICU: A Retrospective Cohort Study
Source: Nurs Crit Care. 2026 Aug 5;31(5):e70600. doi: 10.1111/nicc.70600 (PMC13442041; doi:10.1111/nicc.70600)
Supplement: Supplementary file 2 [file NICC-31-0-s002.docx]

**Supplementary Material S2 – Supplementary Table.** Frequency of the nursing diagnoses identified (n = 904).

| **Nursing Diagnosis** | **Frequency (%)** | **95% CI** |
| --- | --- | --- |
| Risk for infection (00004) | 729 (80.64) | (77.94 - 83.09)† |
| Risk for adult pressure injury (00304)‡ | 433 (47.89) | (44.66 - 51.16)† |
| Acute pain (00132) | 344 (38.05) | (34.94 - 41.26)† |
| Impaired tissue integrity (00044) | 322 (35.62) | (32.56 - 38.80)† |
| Risk for falls (00155) | 321 (35.51) | (32.46 - 38.68)† |
| Risk for aspiration (00039) | 301 (33.29) | (30.30 - 36.43)† |
| Risk for unstable blood glucose (00179) | 247 (27.32) | (24.52 - 30.32)† |
| Decreased cardiac output (00029) | 231 (25.55) | (22.82 - 28.5)† |
| Bathing self-care deficit (00108) | 187 (20.69) | (18.17 - 23.45)† |
| Risk for venous thromboembolism (00268) | 133 (14.71) | (12.55 - 17.18)† |
| Ineffective breathing pattern (00032) | 123 (13.61) | (11.52 - 16.00)† |
| Toileting self-care deficit (00110) | 113 (12.50) | (10.50 - 14.82)† |
| Ineffective peripheral tissue perfusion (00204) | 109 (12.06) | (10.09 - 14.35)† |
| Risk for bleeding (00206) | 95 (10.51) | (8.67 - 12.68)† |
| Ineffective tissue perfusion (specify type: renal, cerebral, cardiopulmonary, gastrointestinal, peripheral) (00024) | 87 (9.62) | (7.86 - 11.73)† |
| Impaired spontaneous ventilation (00033) | 87 (9.62) | (7.86 - 11.73)† |
| Acute confusion (000128) | 86 (9.51) | (7.76 - 11.61)† |
| Impaired gas exchange (00030) | 65 (7.19) | (5.67 - 9.07)† |
| Ineffective protection (00043) | 62 (6.86) | (5.38 - 8.71)† |
| Risk for imbalanced fluid volume (00025) | 61 (6.75) | (5.28 - 8.58)† |
| Impaired skin integrity (00046) | 49 (5.42) | (4.11 - 7.11)† |
| Risk for electrolyte imbalance (00195) | 48 (5.31) | (4.02 - 6.98)† |
| Risk for peripheral neurovascular dysfunction (00086) | 40 (4.42) | (3.25 - 5.98)† |
| Ineffective airway clearance (00031) | 37 (4.09) | (2.97 - 5.60)† |
| Anxiety (00146) | 28 (3.09) | (2.13 - 4.46)† |
| Risk for acute confusion (00173) | 28 (3.09) | (2.13 - 4.46)† |
| Activity intolerance (00092) | 27 (2.99) | (2.04 - 4.33)† |
| Impaired physical mobility (00085) | 22 (2.43) | (1.59 - 3.68)† |
| Nausea (00134) | 20 (2.21) | (1.42 - 3.41)† |
| Delayed surgical recovery (00100) | 17 (1.88) | (1.15 - 3.01)† |
| Chronic confusion (00129) | 16 (1.77) | (1.07 - 2.88)† |
| Excess fluid volume (00026) | 15 (1.66) | (0.98 - 2.74)† |
| Risk for unstable blood pressure (00267) | 15 (1.66) | (0.98 - 2.74)† |
| Adult pressure injury (00312) | 14 (1.55) | (0.90 - 2.61)† |
| Risk for impaired skin integrity (00047) | 13 (1.44) | (0.82 - 2.47)† |
| Chronic pain (00133) | 12 (1.33) | (0.73 - 2.33)† |
| Risk for ineffective cerebral tissue perfusion (00201) | 12 (1.33) | (0.73 - 2.33)† |
| Risk for shock (205) | 12 (1.33) | (0.73 - 2.33)† |
| Impaired urinary elimination (00016) | 11 (1.22) | (0.65 - 2.19)† |
| Risk for ineffective renal perfusion (00203) | 10 (1.11) | (0.57 - 2.05)† |
| Risk for decreased cardiac tissue perfusion (00200) | 9 (1.00) | (0.49 - 1.91)† |
| Feeding self-care deficit (00102) | 8 (0.89) | (0.42 - 1.77)† |
| Risk for impaired liver function (00178) | 8 (0.89) | (0.42 - 1.77)† |
| Disorder of sensory perception (00122) | 7 (0.77) | (0.34 - 1.62)† |
| Risk for imbalanced body temperature (00005) | 6 (0.66) | (0.27 - 1.48)† |
| Hyperthermia (00007) | 6 (0.66) | (0.27 - 1.48)† |
| Impaired walking (00088) | 6 (0.66) | (0.27 - 1.48)† |
| Diarrhea (00013) | 5 (0.55) | (0.20 - 1.33)† |
| Imbalanced nutrition: less than body  requirements (00002) | 5 (0.55) | (0.20 - 1.33)† |
| Dysfunctional ventilatory weaning  response (00034) | 4 (0.44) | (0.13 - 1.18)† |
| Impaired bed mobility (00091) | 4 (0.44) | (0.13 - 1.18)† |
| Impaired swallowing (00103) | 3 (0.33) | (0.06 - 1.02)† |
| Risk for constipation (00015) | 3 (0.33) | (0.06 - 1.02)† |
| Risk for activity intolerance (00094) | 3 (0.33) | (0.06 - 1.02)† |
| Ineffective health maintenance (00099) | 3 (0.33) | (0.06 - 1.02)† |
| Interrupted breastfeeding (00105) | 2 (0.22) | (0.01 - 0.86)† |
| Bowel incontinence (00014) | 2 (0.22) | (0.01 - 0.86)† |
| Risk for ineffective peripheral tissue  perfusion (00228) | 2 (0.22) | (0.01 - 0.86)† |
| Urinary retention (00023) | 2 (0.22) | (0.01 - 0.86)† |
| Deficient fluid volume (00027) | 2 (0.22) | (0.01 - 0.86)† |
| Impaired oral mucous membrane integrity (00045) | 2 (0.22) | (0.01 - 0.86)† |
| Ineffective thermoregulation (00008) | 2 (0.22) | (0.01 - 0.86)† |
| Chronic sorrow (00137) | 1 (0.11) | (0.00 - 0.69)† |
| Stress urinary incontinence (00017) | 1 (0.11) | (0.00 - 0.69)† |
| Stress overload (00177) | 1 (0.11) | (0.00 - 0.69)† |
| Readiness for enhanced self-care (00182) | 1 (0.11) | (0.00 - 0.69)† |
| Urge urinary incontinence (00019) | 1 (0.11) | (0.00 - 0.69)† |
| Dysfunctional gastrointestinal motility (00196) | 1 (0.11) | (0.00 - 0.69)† |
| Risk for dysfunctional gastrointestinal motility (00197) | 1 (0.11) | (0.00 - 0.69)† |
| Functional urinary incontinence (00020) | 1 (0.11) | (0.00 - 0.69)† |
| Risk for ineffective gastrointestinal perfusion (00202) | 1 (0.11) | (0.00 - 0.69)† |
| Risk for allergy reaction (00217) | 1 (0.11) | (0.00 - 0.69)† |
| Chronic pain syndrome (00255) | 1 (0.11) | (0.00 - 0.69)† |
| Risk for deficient fluid volume (00028) | 1 (0.11) | (0.00 - 0.69)† |
| Risk for injury (00035) | 1 (0.11) | (0.00 - 0.69)† |
| Risk for suffocation (00036) | 1 (0.11) | (0.00 - 0.69)† |
| Impaired verbal communication (00051) | 1 (0.11) | (0.00 - 0.69)† |
| Hypothermia (00006) | 1 (0.11) | (0.00 - 0.69)† |
| Ineffective health management (00078) | 1 (0.11) | (0.00 - 0.69)† |
| Fatigue (00093) | 1 (0.11) | (0.00 - 0.69)† |
| Insomnia (00095) | 1 (0.11) | (0.00 - 0.69)† |

†Agresti-Coull method. ‡Some patients were diagnosed with “Risk for pressure ulcer (00249)” and others with “Risk for adult pressure injury (00304)”, the latter being the updated term in the 12th edition of the NANDA-I classification (2021-2023). For this study, both diagnoses were considered equivalent and analyzed as a single diagnosis. CI: Confidence interval.
